# Returns to food and agricultural R&D investments in Sub-Saharan Africa, 1975–2014

**DOI:** 10.1016/j.foodpol.2016.09.009

**Published:** 2016-12

**Authors:** Philip G. Pardey, Robert S. Andrade, Terrance M. Hurley, Xudong Rao, Frikkie G. Liebenberg

**Affiliations:** a Department of Applied Economics, University of Minnesota, St. Paul, MN 55108, USA; b Business Economics, Wageningen UR, Wageningen 6706 KN, Netherlands; c Department of Agricultural Economics, Extension and Rural Development, University of Pretoria, Pretoria 0002, South Africa

**Keywords:** Internal rates of return, Benefit-cost ratios

## Abstract

Research-enabled growth in agricultural productivity is pivotal to sub-Saharan Africa’s overall economic growth prospects. Yet, investments in research and development (R&D) targeted to many national food and agricultural economies throughout Africa are fragile and faltering. To gain insight into what could be driving this trend, this article updates, summarizes and reassesses the published evidence on the returns to African agricultural R&D. Based on a compilation of 113 studies published between 1975 and 2014 spanning 25 countries, the reported internal rates of return (*IRRs*) to food and agricultural research conducted in or of direct consequence for sub-Saharan Africa averaged 42.3%py. In addition to the 376 *IRR* estimates, the corresponding 129 benefit-cost ratios (*BCRs*) averaged 30.1. Most (96.5%) of the returns-to-research evaluations are of publicly performed R&D, and the majority (87.6%) of the studies were published in the period 1990–2009. The large dispersion in the reported *IRRs* and *BCRs* makes it difficult to discern meaningful patterns in the evidence. Moreover, the distribution of *IRRs* is heavily (positively) skewed, such that the median value (35.0%py) is well below the mean, like it is for research done elsewhere in the world (mean 62.4%py; median 38.0%py). Around 78.5% of the evaluations relate to the commodity-specific consequences of agricultural research, while 5.5% report on the returns to an “all agriculture” aggregate. The weight of commodity-specific evaluation evidence is not especially congruent with the composition of agricultural production throughout Africa, nor, to the best that can be determined, the commodity orientation of public African agricultural R&D.

## 1 Introduction

There is a widespread and long-standing consensus that research-enabled growth in agricultural productivity is pivotal to sub-Saharan Africa’s (SSA) overall economic growth prospects, especially in the decades ahead when agriculture will continue to constitute a large (albeit likely declining) share of economic activity and source of employment for most countries in the region (Eicher, 1985, Lipton, 1988, Alston and Pardey, 2014, World Bank, 2015, ADB, 2015). Notwithstanding this consensus, investments in research and development (R&D) targeted to many national food and agricultural economies throughout Africa are fragile and faltering. Pardey et al. (2016a) reported that in 2011, national investments in food and agricultural R&D (public and private *combined*) throughout SSA constituted just 3.9% ($2.7 billion, 2009 PPP prices) of the corresponding world total investment of $69.3 billion; a decline on the 6.1% SSA share three decades earlier in 1980.^1^ Moreover, 39% (i.e., 17 of a total of 44) of the region’s countries spent less on public food and agricultural R&D in 2011 than they did in 1980, after adjusting for the rising costs of research.

In spite of the economic development importance placed on agricultural R&D investments in SSA, there have been only a handful of efforts to summarize and assess the returns to these investments. Oehmke et al. (1991) found just 12 studies to include in their evaluation of the economic returns to African R&D while Oehmke and Crawford (1996, Table 2) reviewed 15 such studies, 7 of which reported on the returns to maize research for 6 SSA countries. Masters et al. (1998, Tables 1 and 2) tabulated and discussed 31 studies (34 estimates), while Alston et al. (2000, Table 7) included 47 studies reporting 201 estimates of relevance for SSA agriculture in their meta-review of the evidence on the returns to R&D throughout the world. Maredia et al. (2000, Table 11) included 26 studies (27 estimates) that reported returns-to-research estimates for major food crops throughout SSA. In their assessments of the returns to SSA-oriented R&D conducted by CGIAR Consortium (or CG for short) centers, Maredia and Raitzer, 2006, Maredia and Raitzer, 2010 pointed to just 23 studies (of which only a subset of 12 studies reported rates-of-return metrics) documenting the economic impact of CGIAR centers and their national partners in SSA.^2^ In this article we update, summarize and reassess the published evidence on the returns to African agricultural R&D, drawing from the comprehensive assessment of the returns to food and agricultural R&D worldwide reported by Hurley et al. (2016).

## 2 R&D evaluation evidence

Hurley et al. (2016) summarized the evidence on the economic consequences of R&D gleaned from a compilation of 492 published studies spanning the period 1958–2015 that reported 3426 rate-of-return estimates for 78 countries throughout the world.^3^ That compilation constitutes the latest (version 3.0) of the InSTePP (International Science and Technology Practice and Policy center, University of Minnesota) returns-to-research database, which involved a concerted effort to develop a comprehensive account of the published evidence, a substantial share of which takes the form of grey literature.

Version 3.0 of the database builds directly on two prior versions, variants of which underpinned the work reported in Alston et al. (2000) and Hurley et al. (2014). To update the database, Pardey et al. (2016b) conducted a systematic search of the literature using the EconLit, AgEconSearch, JSTOR, RePEc, SSRN, ProQuest, and Google search engines, and also searched listings of the evaluation literature posted on-line by agencies such as the CGIAR, ACIAR, and others that were published through to 2015. References cited in the studies identified through these searches were further reviewed to reveal any additional studies not already in the database. Each of these studies was manually scored for a multitude of attributes using a modified version of the questionnaire developed by Alston et al. (2000), which were subsequently entered into the InSTePP database. In total, Pardey et al. (2016b) added 746 new returns-to-research estimates (or 586 evaluations) and associated study and evaluation details taken from 122 different studies relative to version 2.0 of the database. The evidence presented here concerns research conducted in or of direct consequence for SSA and comes from 113 studies reporting 456 evaluations (16.1% of all the evaluations in the InSTePP version 3.0 collection).^4^ A detailed tabulation of the SSA return-to-research evidence and a complete listing of all the source publications is included in the on-line supplementary material.

### 2.1 Methodological attributes

**Fig. 1:**
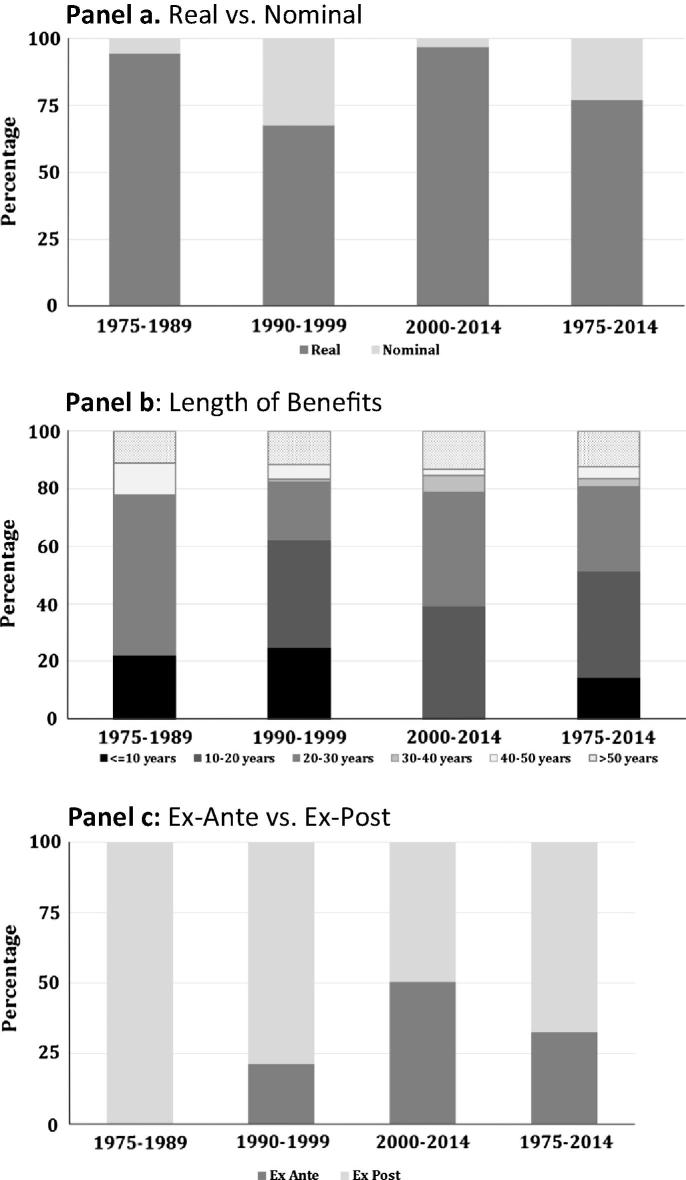
Methodological variations.

Researchers estimating the returns to R&D have a variety of methodological choices to make. The choices made have varied over time and can influence the rate-of-return estimates. While some studies evaluated the nominal costs and benefits of an investment, the majority of the SSA return-to-research evaluations (77.1%) have taken inflation into account by evaluating the relationship between real costs and real benefits (Fig. 1, Panel a). Between 1990 and 1999, studies using nominal values were more common than prior to 1990 or after 1999. This may be attributable to the highly inflationary period of the 1990s and the difficulty in choosing appropriate deflators to estimate the real costs and benefits of research.

Agricultural R&D often produces long-lasting benefits and the length of time over which these benefits are evaluated can influence the estimated rate of return. The average lag length—i.e., the period over which research spending begins and the associated benefit stream ends—for the (finite) research benefit streams in the SSA studies is 19.9 years; ranging from a low of zero to a maximum of 73 years (Fig. 1, Panel b).^5^ In comparison, the average lag length for the (finite) benefit stream in the studies reporting research conducted elsewhere in the world is almost six years longer (25.7 years), ranging from lag lengths of zero to 142 years.

In addition to these and other methodological attributes, evaluations can differ depending on whether they are *ex post* for a completed or *ex ante* of a yet to be performed investment. Although the majority of the SSA evaluations reported in the literature (308 out of 456 evaluations) were *ex post* rather than *ex ante*, a much larger share of the SSA evaluations were *ex ante* (32.5%) compared with those reporting returns to research elsewhere in the world (20.3%). In SSA there is also an increasing emphasis over time on *ex ante* evaluations, perhaps driven by increased up-front accountability demands by research funding agencies, but it may also be a reflection of a continuing lack (if not increasing scarcity) of newly observed data on the uptake and consequence of agricultural technologies throughout SSA.

Social R&D evaluations attempt to evaluate the costs and benefits of an investment accruing to all members of society, while private evaluations focus only on the costs and benefits accruing to a particular societal group. Given the paucity of privately performed food and agricultural R&D in the region (Pardey et al., 2016a) most of the evidence (390 of a total of 456 evaluations) *explicitly* report social returns to R&D, with the social versus private dimensions of the benefits being unspecified in the remaining studies.

## 3 R&D evaluations characterized

**Fig. 2:**
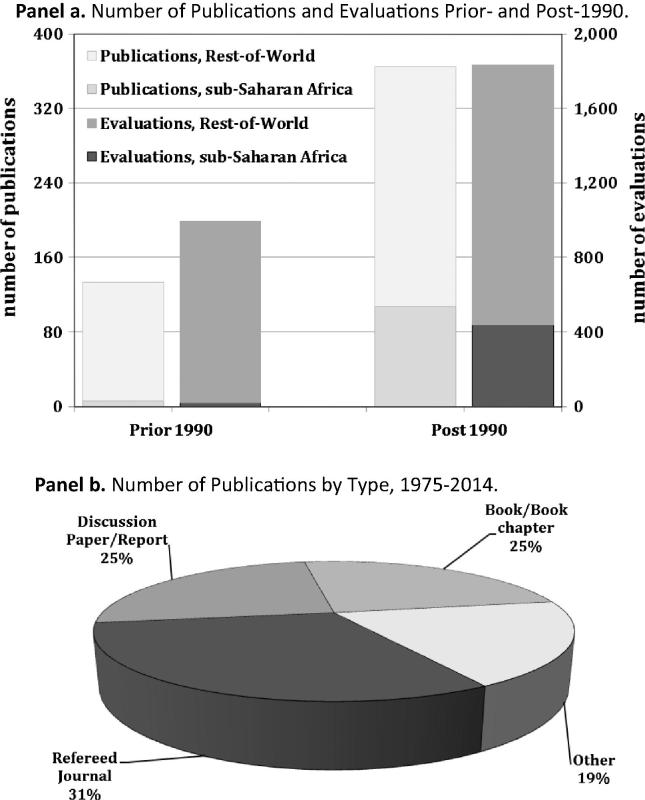
The Published Returns-to-Research Evidence for sub-Saharan Africa, 1975–2014. Notes: The Rest-of-World in Panel a includes all the returns-to-research evaluations reported by Hurley et al. (2016) excluding those designated SSA evaluations. “Other” in Panel b includes graduate dissertations, conference papers and grey literature.

The first reported estimate of the returns to agricultural R&D in SSA we found came almost two decades after the classic 1958 hybrid corn evaluation study by Griliches. It is a study published by Evenson and Kislev in 1975 (chapter 3) on the returns to sugarcane research in South Africa.^6^ The latest study in this SSA compilation was published in 2014. The overwhelming majority of the SSA evidence was published after 1990: just 6 studies (5.3% of the SSA total, and just 4.5% of the corresponding studies published worldwide) were published prior to 1990 (Fig. 2, Panel a). However, the post-1990 pattern of published evaluation evidence has been uneven. The number of published studies surged during the 1990s, totaling 69 studies. Thereafter, evaluation interest seems to have waned, with 30 studies published in the 2000s, and only 8 published studies thereafter. Despite the apparent emphasis on the increased accountability of (research and related) investments in SSA agriculture in more recent years (e.g., Bill and Melinda Gates Foundation, 2015, USAID, 2015), there has not been increased evaluation evidence generated, at least not evidence about the economic returns to those investments.

Around 30.9% of the SSA evaluations were published in refereed journals, about the same share as the worldwide compilation of agricultural R&D evaluation studies (Hurley et al., 2016). The remaining evaluations were published in a variety of outlets, including books, theses, reports or grey literature (Fig. 2, Panel b).

**Fig. 3:**
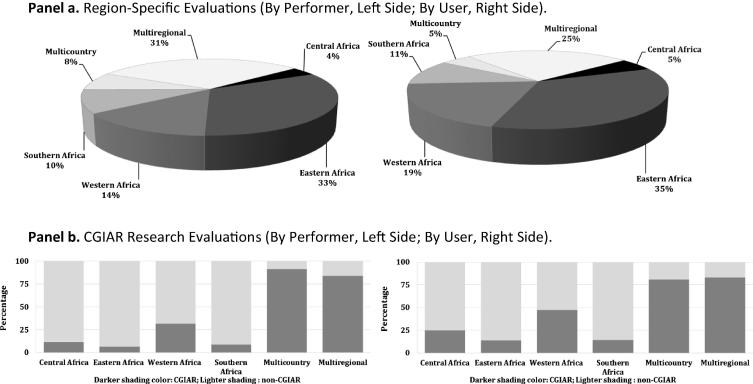
The Geography of Evaluations by Research Performer and User. Notes: In Panels a and b, “by-performer” reports the evaluation evidence in relation to where the research was performed; the “by user” compilation is in terms of where in SSA the results of the research were adopted and used. Countries are grouped according to FAO (2016) regional classifications. The percentages in Panel b indicate the share of each grouping relating to research involving CGIAR centers versus other (often national public) research agencies.

Fig. 3, Panel a gives a regional breakdown of the returns-to-research evidence in terms of where in SSA the research was performed versus where in SSA the results of the research were adopted and used. The evidence spans 25 of the total of 48 SSA countries when reported on a by-performer basis, and 32 countries when reported on a by-user basis. Thus 87% of the region’s 2013 agricultural output and 82% of the region’s poor people (i.e., those living on less than $1.25 per day) are encompassed by the countries included in the by-user group (World Bank, 2015a, World Bank, 2015b). The regional by-performer versus by-user shares are similar, indicating the benefits included in each evaluation are typically limited to those that accrue within the country or region in which the research was undertaken (and consistent with the fact that only 5.5% of the country-specific evaluations paid any explicit attention to research spill-ins or spill-outs). Roughly one-third of the evaluations refer to research done in or impacting Eastern Africa (with studies for Uganda and Zambia accounting for 89 of that region’s 150 internal rates of return, *IRR*, evaluations). One notable aspect of the regional split is the surprisingly small number of evaluation studies for Southern Africa compared with Eastern or Western Africa, even though that part of the continent accounted for 22.5% of the region’s public food and agricultural R&D spending for the period 1975–2011 (Pardey et al., 2016a). A large share of the SSA evaluation evidence (specifically 39.0% of the evidence reported on a by-performer basis, and 29.4% on a by-user basis) consists of African *IRR* results obtained in the context of evaluations that encompassed more than one country (i.e., multi-country studies) or more than one region of the world, one of which was SSA (i.e., multi-regional studies). Moreover, the overwhelmingly dominant share (more than 85.4%) of both these types of (multi-country, multi-regional) studies involved the evaluation of CGIAR research, including CG-specific research or research carried out by the CG in partnership with national agencies throughout the region (Fig. 3, Panel b).

Looking into more detail regarding the agencies carrying out the R&D being evaluated, public organizations make up 96.5% (440 of 456) of the evaluations. Research conducted at universities throughout SSA represents just 3.5% of the public evaluations. Research conducted exclusively by national agencies constituted 50.4% of the public-sector evaluations (or 53.9% if research done jointly with universities and private entities is included). Research conducted exclusively by international agencies accounts for a further 20.7% of this public total, with a further 25.0% of the evaluations being conducted by international agencies joint with national government, university and private partners. CG centers constituted 93.1% of all the evaluations associated with international agencies. A tiny fraction, just 2.9% (i.e., 13 evaluations taken from 9 studies), relate to research carried out by private entities, of which 12 of the 13 evaluations involved joint private and public R&D. The only study that focused exclusively on privately performed R&D is a 2004 study by Nieuwoudt and Nieuwoudt on South African sugarcane research, the same subject matter as the first SSA study by Evenson and Kislev (1975). Other evaluations that involved privately performed R&D (joint with public research conducted by universities, CGIAR centers and government research agencies) include an Ethiopian study of maize research, South African studies of ornamental flowers and wine grape R&D, and Zimbabwean research on cotton and groundnuts. Notably, all but the maize study relate to private research conducted on export-oriented, cash crops rather than food staples.

**Fig. 4:**
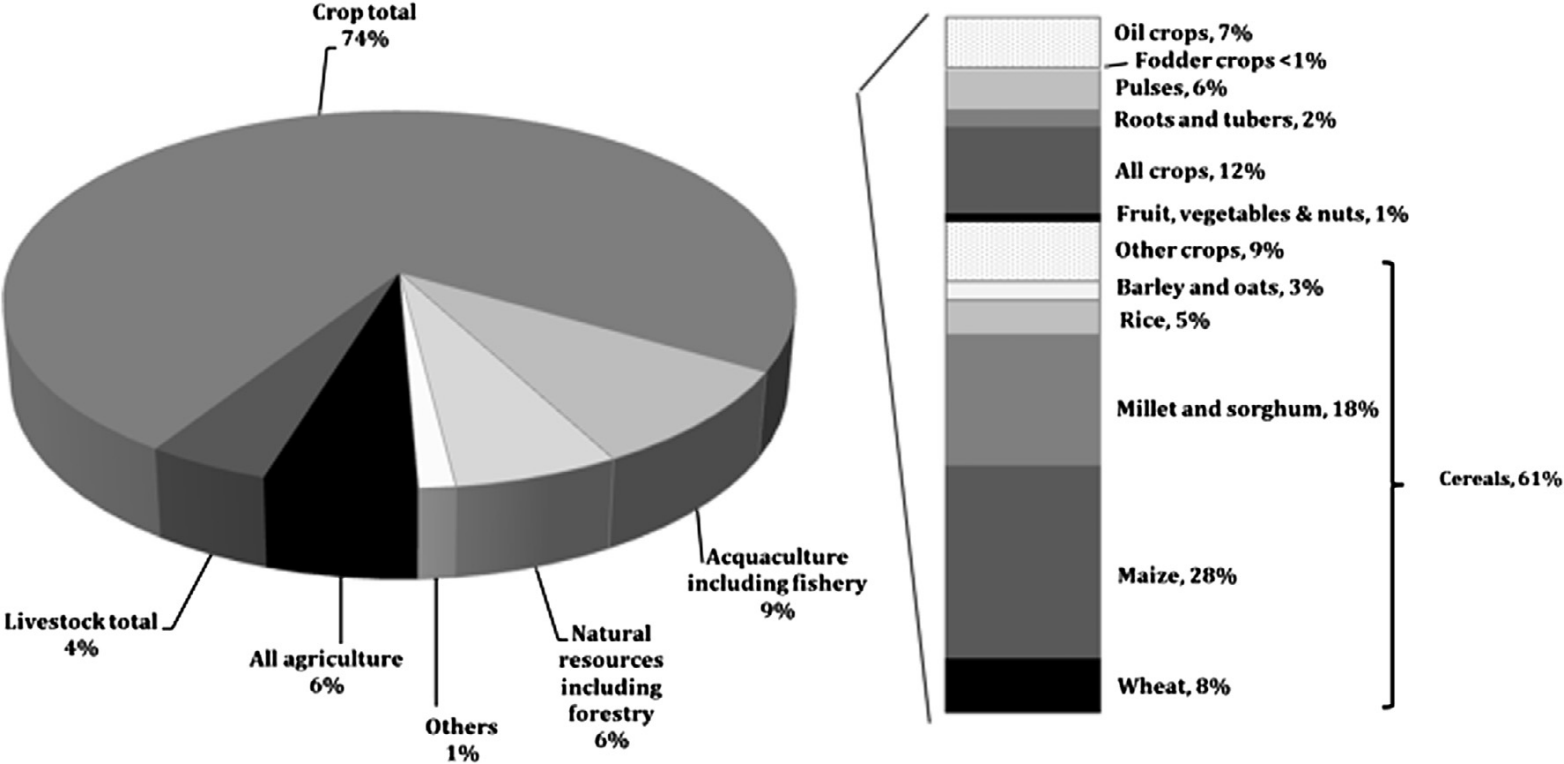
Evaluations by Commodity Categories. Notes: Commodities are grouped into categories according to FAO classifications (see notes to Table 3 for details). To maximize the concordance of the commodity categories presented here with those reported in Table 3, the “Others” category includes joint crop-livestock, agriculture-fishing and agriculture-aquaculture research, plus research on “other trees.” The stacked bar reports various commodity shares within the “Crop total” category.

Just over one third (36.9%) of the evaluations report the returns to research (be it basic or applied R&D), just 2.4% of the evaluations related solely to extension, while the majority of the evaluations (59.9%) report the joint returns to research and extension. The preponderance (74.1%) of the region’s evaluation evidence relates to crop research, which is well in excess of the corresponding 51% share of worldwide studies (Hurley et al., 2016). Within crops, the major focus of the SSA evaluation evidence is on maize, millet and sorghum research. These three crops alone constitute almost half (45.9%) of the crop-related evaluations (Fig. 4). Notably, little evaluation evidence exists for roots and tuber crops like cassava and yams (1.3% of the total returns to research evaluations for SSA), even though those crops constituted 18.0% by value of SSA agricultural output over the past two decades (1994–2013) (FAO, 2015). Similarly, livestock evaluation studies represent a smaller share (4.4%) of all the SSA evaluation studies; substantially less than the 24.3% by value share of the region’s overall agricultural output attributable to livestock products.

## 4 The reported rates of return

The overwhelming majority (82.5%) of the 456 rate-of-return evaluations for SSA from a total of 113 studies report an *IRR* measure. Of the 113 studies, 74.3% report only *IRRs*, 10.7% report only benefit-cost ratios (*BCRs*), and 15.9% report both *IRRs* and *BCRs*. Like the rates of return to research literature generally, the published SSA studies strongly prefer *IRRs* as the means to summarize the stream of benefits and costs associated with agricultural R&D, even though Griliches (1958) expressed a preference for the *BCR*. Hurley et al. (2014) suggested using the modified *IRR*, which is simply another way of writing the *BCR*.

**Fig. 5:**
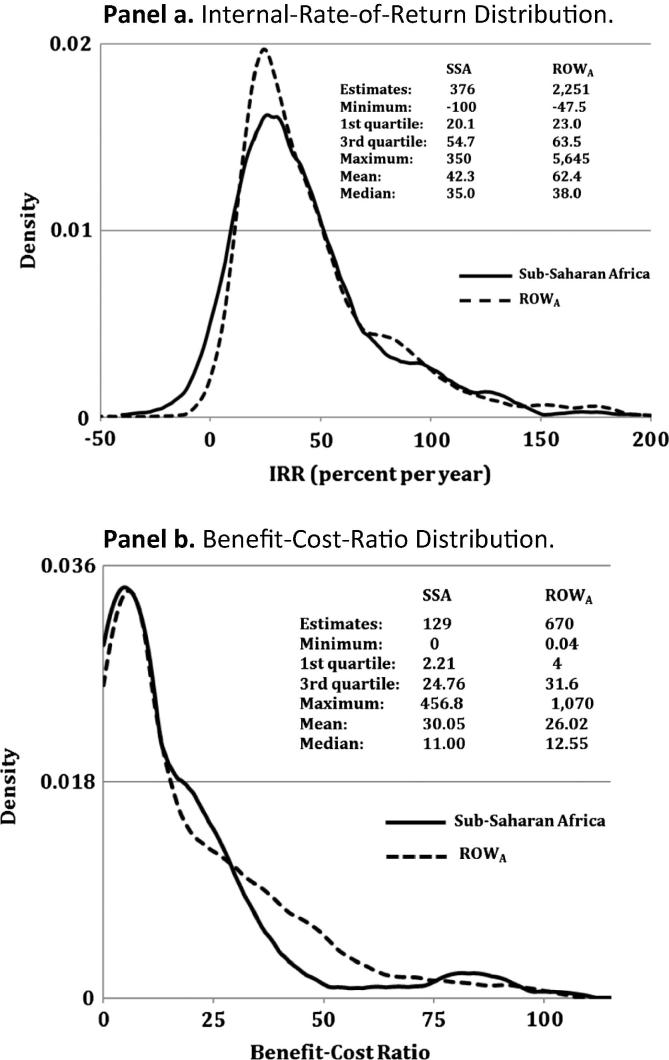
Distribution of Internal-Rate-of-Return and Benefit-Cost-Ratios. Notes: Vertical axis represents relative frequency. For display purposes the plotted distribution was truncated at −50 and 200 in Panel a and at 0 and 110 in Panel b. ROW_A_ refers to the overall number of evaluations excluding those of direct relevance to SSA (see Footnote 3).

Fig. 5, Panel a shows the distribution of *IRRs* and other common statistics for both SSA and the rest-of-the-world (ROW_A_: world excluding evaluations of direct relevance to SSA). Both distributions are skewed to the right, with their mean higher than their median *IRRs*. A Kolmogorov-Smirnov test confirms that the distributions are different at the 10% level of significance, with a substantially lower mean (42.3 versus 62.4%py) and marginally lower median (35.0 versus 38.0%py) *IRRs* reported for SSA compared with elsewhere in the world (Fig. 5, Panel a). While the BCRs in Fig. 5, Panel b would seem to indicate less of a discrepancy between the SSA and ROW_A_ returns-to-research evidence with the mean *BCR* for SSA actually higher than the ROW_A_ (30.1 versus 26.0) and the median *BCR* for SSA is a little lower than ROW_A_ (11.0 versus 12.6), a Kolmogorov-Smirnov test again reveals the distributions are different at the 10% level of significance.

Table 1 provides various summary statistics of the distribution of IRRs reported for each region within SSA. It also reports these same statistics for multi-country and multi-regional research that includes (parts of) SSA. Some of the regional summary statistics need to be taken with a grain of salt. For example, the *IRR* evidence for the Central region spans only two countries and draws from only four studies that report a total of 17 IRR estimates. While the statistics for the Southern region represent a summary of only 44 *IRR* estimates, they are drawn from 19 different studies. The dispersion among regions in terms of the median *IRRs* is much more muted than the means, with the returns to multi-country and multi-regional research tending to be at the upper end of the reported range.

Table 2 groups the *IRR* observations in terms of their various research versus extension orientations. There is some suggestion in this evidence that investments in extensions services, either in and of themselves or coupled with investments in R&D, are especially rewarding. The two extension-related categories have median *IRRs* that exceed the “All studies” median (35.0%py) and are greater than the corresponding medians for the “Applied” and “Basic and Applied” research-only groupings.

Table 3 groups the *IRR* evidence according to its commodity focus. Reiterating the findings from Fig. 4, almost all (86.8%) of the evidence pertains to crop research. The reported returns to livestock and natural resource oriented agricultural R&D are grossly under-represented in this literature, both in relation to the economic value of agricultural output and the orientation of the research itself (data underlying Pardey et al., 2016a). One stand-out entry in this tabulation is the median returns to rice research, which are more than double the overall median returns to R&D. The comparatively limited amount of evaluation evidence on rice research cautions against putting too much stock in this finding. The returns to maize research are at the upper end of the range, and are substantially larger than the average or median return to research on small grain cereals such as millet and sorghum.

**Fig. 6:**
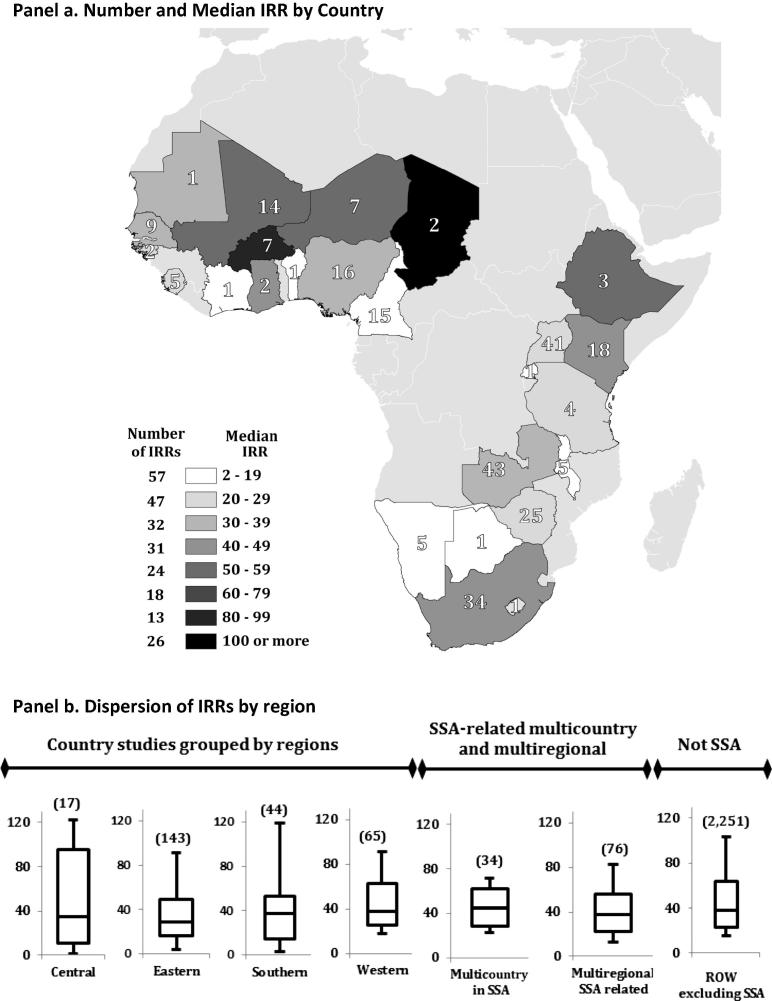
The African geography of the returns to research evidence. Notes: Panel a displays the number of IRR estimates per country for the period 1975–2014. The shading indicates the range within which the median IRR for each country falls. The horizontal bar within the box and whiskers plots in Panel b indicates the median, the lower and upper ends of each box the 25th and 75th percentile respectively, with the lower and upper ends of the whiskers being the 10th and 90th percentile respectively.

Fig. 6, Panel a gives a mapped representation of the number of IRR evaluations and corresponding median *IRR* by country. There is little concordance between the geographical distribution of the rate-of-return evidence and the value of agricultural output. For example, Nigeria with 36.1% of SSA’s 2014 agricultural output (by value) accounts for just 4.3% of the region’s *IRRs*, whereas Zambia with less than one percent of the value of output constitutes 11.4% of the reported *IRRs*. Moreover, just five of the region’s 48 countries (Kenya, South Africa, Uganda, Zambia, and Zimbabwe) account for 42.8% of the published *IRRs*.

The box-whiskers plots in Fig. 6, Panel b provide more detail on the nature of the dispersion in *IRR* estimates within the different regions in SSA. The horizontal bar within each box indicates the median *IRR*, and the upper and lower values of the whiskers represent the 90th and 10th percentile reported *IRR* in each region. The preponderance of the evidence within each region is reasonably tightly clustered around the mean, except for the 17 evaluations in central Africa where the dispersion around the median is more pronounced.

## 5 Conclusion

The wide dispersion in the reported *IRRs* for SSA and elsewhere in the world makes it difficult to discern meaningful patterns in the evidence. Moreover, the skewed nature of the *IRR* distributions indicates that the median is a more informative measure of central tendency than the mean, although there are indications that the mean *IRR* for SSA is meaningfully different from the rest-of-world average (i.e., the world excluding Africa). Hurley et al. (2016) reported a worldwide average and median IRR to research (and extension) of 59.6 and 37.5%py respectively. More specifically, the corresponding SSA figures are 42.3 and 35%py respectively, compared with 62.5 and 38%py for the concordant rest-of-world figures.

This survey raises questions as to the representativeness of the African returns-to-research evidence. The commodity focus of this evidence is not especially congruent with either the composition of African agricultural production nor, to the best we can ascertain, the commodity emphasis of public agricultural R&D throughout the region (data underlying Pardey et al., 2016a). In both instances the rate-of-return evidence is over representative of crops research (and thus under-representative of livestock research). It also gives short shrift to research related to non-cereal crops, especially root crops, bananas and plantains, which are important from both a production and a consumption perspective throughout substantial parts (but by no means all) of Africa.

This study raises further questions as to the utility of this evidence in drawing general conclusions about the overall returns to investments in African agricultural R&D. The portfolio of available *IRR* and *BCR* studies likely represents a complex combination of factors, including the resources available to carry out such studies, the availability of data—an especially problematic issue for SSA where relevant data on technology adoption and its productivity consequence are especially limited (see, for example, Walker et al., 2014)—, and the scope of the methodological toolkit to enable such studies. To the extent the reported IRRs and BCRs are biased towards studies of “successful” research, the body of evidence would give an upwardly biased representation of the returns to research in the region. Offsetting these concerns is the fact that the median of the reported *IRRs* for the “all agriculture” portfolio of studies is 39.0% per year, slightly above the median *IRR* for the commodity specific evidence summarized in Table 3, which is 35.1% per year.

Relative to elsewhere in the world, a much larger share of the returns-to-research evidence for SSA is more speculative (ex ante) evidence on the prospective returns to research yet to be undertaken (or completed). Moreover, an ever-larger share of the SSA returns-to-research estimates are shifting in favor of ex ante rather than ex post assessments of the benefits already realized from past R&D endeavors. To the extent this change in the balance of the research evaluation evidence reflects increasingly scarce information on the uptake or (productivity) consequences attributable to research induced technical change in more recent years, it raises concerns about the growth prospects for African agricultural over the decades ahead. Certainly the research investment evidence (e.g., Pardey et al., 2016a) is particularly worrisome. It suggests that many countries throughout SSA are failing to sustain the long-run commitments to investments in R&D (and associated educational and science-based regulatory capabilities) required to develop the local innovation and institutional capacities that have been pivotal to the agricultural productivity performance of countries elsewhere in the world. This is in spite of the growing body of *IRR* and *BCR* estimates reported here, which suggests that overall, governments throughout the region are continuing to underinvest in research directed to their food and agricultural sectors.

## Figures and Tables

**Table 1:** Reported internal rate of return estimates by geographic region of research performer.

	Number of			Central tendency			Range			
	Countries	Estimates	Publications	Mean	Median	Standard deviation	Minimum	Maximum	5th Percentile	95th Percentile
	*(count)*			*(percent per year)*			*(percent per year)*			
Central	2	17	4	52.7	35.0	57.1	−2.3	188.0	−2.3	188.0
Eastern	8	140	33	37.0	28.5	45.3	−100.0	350.0	−9.94	106.0
Southern	4	44	19	43.8	37.5	41.7	−12.0	170.0	2.0	135.0
Western	11	65	22	47.5	38.0	31.7	−6.0	136.0	13.0	123.0
Multi-country		34	8	44.9	44.5	22.4	4.0	95.0	5.0	84.0
Multi-regional		76	22	43.3	37.5	27.0	0.0	132.3	8.3	97.4
All studies		376	102	42.3	35.0	38.5	−100.0	350.0	2.0	110.3

Notes: Table excludes information from 11 publications that report only BCRs.

**Table 2:** Reported internal rate of return estimates by R&D orientation.

	Number of		Central tendency			Range			
	Estimates	Publications	Mean	Median	Standard deviation	Minimum	Maximum	5th Percentile	95th Percentile
	*(count)*		*(percent per year)*			*(percent per year)*			
Applied	8	7	29.5	30.5	8.5	14.0	40.0	14.0	40.0
Basic and Applied	132	23	35.8	31.5	30.5	−56.6	116.6	−7.0	99.6
Research and Extension	224	68	43.8	37.7	36.6	−100.0	188.0	4.3	122.5
Extension	9	5	95.9	86.0	103.8	7.0	350.0	7.0	350.0
Other	3	3	89.7	88.0	67.3	44.0	167.0	44.0	167.0
All studies	376	102	42.3	35.0	38.5	−100.0	350.0	2.0	110.3

Notes: “Other” includes IRR evaluations that fall outside the categories identified in this table.

**Table 3:** Reported internal rate of return estimates by commodity focus.

	Number of		Central tendency			Range			
	Estimates	Publications	Mean	Median	Standard deviation	Minimum	Maximum	5th Percentile	95th Percentile
	*(count)*		*(percent per year)*			*(percent per year)*			
Crop total	329	84	42.8	35.1	38.6	−100.0	350.0	4.0	106.2
Cereals	211	45	41.7	35.0	34.9	−100.0	135.0	4.0	106.2
Maize	93	20	48.6	43.0	42.0	−100.0	135.0	−22.8	113.9
Millet and Sorghum	62	13	33.5	24.1	28.9	−2.3	122.5	5.0	95.0
Rice	17	4	59.7	74.8	31.9	17.9	102.0	17.9	102.0
Wheat	30	8	30.0	24.0	15.2	3.0	57.0	7.0	54.0
Oil crops	24	5	26.3	25.7	18.2	−12.3	59.0	−3.4	50.0
Pulses	20	10	46.8	42.4	35.9	4.7	132.3	6.5	113.6
Other crops	29	12	37.7	33.6	41.9	−14.3	188.0	−7.4	119.0
Livestock total	14	6	36.9	39.5	16.6	−2.0	55.0	−2.0	55.0
All agriculture	25	7	44.9	39.0	49.8	−12.0	170.0	−7.0	145.0
All studies	376	102	42.3	35.0	38.5	−100.0	350.0	2.0	110.3

Notes: Studies grouped according to FAO commodity classification standards at www.fao.org/waicent/faoinfo/economic/faodef/faodefe.htm; Cereals include barley, maize, millet, rice, sorghum, sorghum/millet and wheat; Fruit, Vegetables & Nuts include apple, banana, beans, cashew nuts, chilies, citrus, cole crops, cucurbit, fruit/nut, guava, leafy vegetables, mango, melon, onion, pineapple, plantain, stone fruits, and tomato; Poultry include poultry; Other Livestock include beef, dairy, dairy and beef, goat, sheep, sheep/goats, buffalo, cattle, other livestock, pork and swine; Natural Resources include forestry and natural resources; All Agriculture include all agriculture.

## Footnotes

1 The $69.3 billion agGERD (gross domestic expenditure on (public and private) food and agriculture R&D) is net of spending by countries of the former Soviet Union and Eastern Europe (FSU&EE) for which Pardey et al. (2016a) have more limited data. Adding in Pardey et al.’s (2016b) unpublished satellite estimates of 2011 agGERD spending by FSU&EE countries puts the corresponding global total at $71.2 billion.

2 For a listing of, and further details on, the CGIAR centers see www.cgiar.org. See also Jahnke et al. (1987) for an earlier review of the impact of agricultural research in tropical Africa. Though comprehensive in scope, the report contained few formal returns-to-research estimates.

3 To tally the number of countries we counted countries in which the evaluated research was performed as distinct from counting countries in which the research was used. Some of the entries in the InSTePP database are tagged as “multi-national” (i.e., more than one country), but in forming the country count reported here we retained the multi-national designation and did not count each of the individual countries included in the multi-national aggregates. Moreover, version 3.0 of the database contains 2827 evaluations, wherein a single evaluation (of a particular technology, project, or program) within a given study may report either an *IRR* or a *BCR* estimate, or both. In this paper we refer to both evaluations and estimates, of which 505 estimates report on research conducted in or of direct consequence for SSA.

4 In scoring these studies, an evaluation report was deemed of direct relevance for SSA if the research was either carried out or utilized within the region (or both) or was directly targeted to SSA as revealed by explicit reference to the region (or countries therein) in the report.

5 These data exclude the 401 evaluations that assumed benefits accrued over an infinite time horizon.

6 The sugarcane research conducted in South Africa consisted mainly of screening and selecting varieties developed or discovered elsewhere (especially from India and Java) undertaken by the grower funded South African Sugar Association Experiment Station (SASEX), which was established at Mount Edgecombe in 1925 (Evenson et al., 1970, pp. 423 and 427; Donovan, 1997).
