# Supplementary material for: Returns to food and agricultural R&D investments in Sub-Saharan Africa, 1975–2014
Source: Food Policy. 2016 Dec;65:1–8. doi: 10.1016/j.foodpol.2016.09.009 (PMC5176338; doi:10.1016/j.foodpol.2016.09.009)
Supplement: Supplementary data 1 [file mmc1.pdf]

Supplementary Material

Returns to Food and Agricultural R&D Investments in Sub-Saharan Africa, 1975-2014

October 18, 2016

Philip G. Pardey, Robert S. Andrade Terrance M. Hurley, Xudong Rao and Frikkie G. Liebenberg

*Pardey and Hurley are Professors and Andrade a Graduate Research Assistant in the Department of Applied Economics at the University of Minnesota. Rao is a lecturer in the Business Economics group at Wageningen UR, and Liebenberg is a Senior Lecturer in the Department of Agricultural Economics, Extension and Rural Development, University of Pretoria. They are all affiliated with the University of Minnesota's International Science and Technology Practice and Policy (InSTePP) Center. The authors thank Connie Chan-Kang, Michelle Hallaway, and Louise Letnes for their excellent research assistance. This paper was prepared with primary support from the CGIAR's Standing Panel on Impact Assessment, with additional support from the University of Minnesota's Agricultural Experiment Station (Project Nos. MIN-14-061 and MIN-14-034), the Centro Internacional de Agricultura Tropical (CIAT), and the Bill and Melinda Gates Foundation by way of the HarvestChoice project.

Table S-1: African Related Research Evaluation Evidence

Authors	Year	Number of		Internal Rate of Return			Benefit-Cost Ratio		
		Evaluations[a]	Estimates[a]	Average	Minimum	Maximum	Average	Minimum	Maximum
Evenson, R., and Kislev, Y.	1975	2	2	34.5	29.0	40.0			
Abidogun, A.	1978	2	2	35.5	34.0	37.0			
Evenson, R., and Flores-Moya, P.	1978	11	22	79.7	32.0	102.0	58.0	8.0	146.0
Evenson, R.	1987	1	1	35.0	35.0	35.0			
Norgaard, R.	1988	1	1				149.0	149.0	149.0
Schwartz, L., Sterns, J., and Oehmke, J.	1989	1	1	63.0	63.0	63.0			
Karanja, D.	1990	2	2	54.5	40.9	68.1			
Mazzucato, V.	1991	2	2	59.0	58.0	60.0			
MacMillan, J., Mudimu, G., MacRobert, J., Rugube, L., Guveya, E., and Mutemeri, I.	1992	1	2	22.0	22.0	22.0	1.4	1.4	1.4
Thirtle, C., Atkins, J., Bottomley, P., Gonese, N., and Goverch, J.	1992	1	1	54.0	54.0	54.0			
Bindlish, V., and Evenson, R.	1993	2	2	201.0	52.0	350.0			
Bindlish, V., Evenson, R., and Gbetibouo, M.	1993	4	4	101.0	86.0	136.0			
Howard, J., Chitalu, G., and Kalonge, S.	1993	12	12	57.3	-100.0	110.3			
Mazzucato, V., and Ly, S.	1993	1	1	10.0	10.0	10.0			
Schwartz, L., Sterns, J., and Oehmke, J.	1993	3	3	63.0	31.0	92.0			
Thirtle, C., Atkins, J., Bottomley, P., Gonese, N., Goverch, J., and Khatri, Y.	1993	1	1	43.0	43.0	43.0			
Thirtle, C., Von Bach, H., and Van Zyl, J.	1993	3	3	136.0	128.0	145.0			
Boughton, D., and De Frahan, B.	1994	10	10	81.8	38.0	135.0			
Byerlee, D., and Traxler, G.	1994	4	4	45.5	25.0	54.0			
Howard, J.	1994	6	6	88.3	42.1	116.6			
Kupfuma, B.	1994	4	4	44.4	43.5	46.5			
Laker-Ojok, R.	1994	19	19	25.8	-12.3	49.0			
Laker-Ojok, R.	1994	20	20	10.0	-56.6	35.8			

Table S-1 – Continued

Authors	Year	Number of		Internal Rate of Return			Benefit-Cost Ratio		
		Evaluations[a]	Estimates[a]	Average	Minimum	Maximum	Average	Minimum	Maximum
Sanders, J.	1994	1	1	74.0	74.0	74.0			
Smale, M., and Heisey, P.	1994	4	4	34.8	4.0	63.0			
Sterns, J., and Bernsten, R.	1994	5	5	6.6	-2.3	15.5			
Anandajayaskeram, P., Martella, D., Sanders, J., and Kuffuma, B.	1995	12	12	20.8	4.3	27.6			
Byerlee, D., and Traxler, G.	1995	4	4	40.0	23.0	52.0			
Lubulwa, G.	1995	1	1	12.8	12.8	12.8			
Lubulwa, G., Gwaze, D., Clarke, J., Milimo, P., and Mulatya, J.	1995	3	3	12.9	0.0	26.8			
Lubulwa, G., Wafula, B., Craswell, E., Willet, I., and Davis, J.	1995	1	1	20.4	20.4	20.4			
Mudhara, M., Anandajayasekeram, P., Kupfuma, B., and Mazhangara, E.	1995	4	4	44.8	42.0	47.2			
Ouedraogo, S., Illy, L., and Lompo, F.	1995	1	1	78.1	78.1	78.1			
Seck, P., Sidibe, M., and Beye, A.	1995	3	3	34.5	32.9	37.0			
Tre, J.-P.	1995	4	4	19.5	17.9	21.4			
Aghib, A.	1996	6	6	55.9	45.0	66.4			
Akgundor, S., Makanda, D., and Oehmke, J.	1996	1	1	14.0	14.0	14.0			
Arnade, C., Khatri, Y., Schimmelpfennig, D., Thirtle, C., and Van Zyl, J.	1996	3	3	90.7	44.0	170.0			
Chisi, M., Anandajayaskeram, P., Martella, D., Ahmed, M., and Mwape, M.	1996	17	17	18.5	10.2	25.2			
Howard, J., and Mungoma, C.	1996	7	7	73.9	42.1	96.0			
Karanja, D.	1996	4	4	49.8	39.0	60.8			
Khatri, Y., and Thirtle, C.	1996	1	1	44.3	44.3	44.3			
Makanda, D., and Oehmke, J.	1996	2	2	7.5	3.0	12.0			
Njomaha, C., Adamou, A., and Yapi, A.	1996	9	9	56.2	10.9	122.5			
Oudraogo, S., and Illy, L.	1996	1	1	7.0	7.0	7.0			
Rueda, J., Elwell, P., Walker, T., Soto, M., Bicamumpaka, M., and Berrios, D.	1996	1	1	84.0	84.0	84.0			

Table S-1 – Continued

Authors	Year	Number of		Internal Rate of Return			Benefit-Cost Ratio		
		Evaluations[a]	Estimates[a]	Average	Minimum	Maximum	Average	Minimum	Maximum
Yallah, N.	1996	1	1	188.0	188.0	188.0			
Yapi, A., Kergna, A., Debrah, S., Sidibe, A., and Sanogo, O.	1996	2	2	60.0	50.0	70.0			
Anandajayasekeram, D., Martella, D., Sanders, J., and Kupfuma, B.	1997	2	2	9.5	5.0	14.0			
Berlin, R.	1997	2	2	34.1	30.6	37.6			
Bindlish, V., and Evenson, R.	1997	1	1	28.0	28.0	28.0			
Edwin, J., and Masters, W.	1997	1	1	34.1	34.1	34.1			
Isinika, A.	1997	1	1	33.2	33.2	33.2			
Mazhangara, E., Anandajayasekeram, P., Mudhara, M., Martella, D., and Murata, M.	1997	1	1	59.0	59.0	59.0			
Moshi, A., Anandajayasekeram, P., Kaliba, A., Martella, D., Mwangi, W., and Shao, F.	1997	2	2	21.0	19.0	23.0			
Murata, M., Anandajayasekeram, P., Mudhara, M., Martella, D., and Mazhangara, E.	1997	2	2	9.0	6.0	12.0			
Niederwieser, J.	1997	2	2	3.0	2.0	4.0			
Oudraogo, S., and Bertelsen, M.	1997	1	1	52.7	52.7	52.7			
Seidi, S.	1997	1	1	26.0	26.0	26.0			
Townsend, R., Van Zyl, J., and Thirtle, C.	1997	1	1	28.8	28.8	28.8			
Bua, A., Acola, G., Adupa, R., Otim-Nape, G., Baguma, Y., and Sserunkuma, D.	1998	1	2	167.0	167.0	167.0	18.2	18.2	18.2
Mokoena, M.	1998	8	8	33.3	-2.0	54.0			
Townsend, R., and Van Zyl, J.	1998	1	1	40.0	40.0	40.0			
Wessels, J., Anandajayasekeram, P., Van Rooyen, C., Marasas, C., Littlejohn, G., and Coetzee, C.	1998	1	1	7.7	7.7	7.7			
Byerlee, D., and Traxler, G.	1999	2	2	37.5	23.0	52.0			
Elbasha, E., Thornton, P., and Tarawali, G.	1999	1	2	38.0	38.0	38.0	3.3	3.3	3.3

Table S-1 – Continued

Authors	Year	Number of		Internal Rate of Return			Benefit-Cost Ratio		
		Evaluations[a]	Estimates[a]	Average	Minimum	Maximum	Average	Minimum	Maximum
Kristjanson, P., Rowlands, J., Swallow, B., Kruska, R., and De Leeuw, P.	1999	1	2	33.0	33.0	33.0	34.0	34.0	34.0
Kristjanson, P., Zerbini, E., Rao, K., Kiresua, V., and Hofs, P.	1999	1	2	28.0	28.0	28.0	15.0	15.0	15.0
Marasas, C.	1999	2	2	24.2	21.6	26.8			
Maredia, M., and Byerlee, D.	1999	14	14	24.9	7.0	43.0			
Mokoena, M., Townsend, R., and Kirsten, J.	1999	2	2	47.5	44.0	51.0			
Mukhebi, A., Chamboko, T., O'Callaghan, C., Peter, T., Kruska, R., and Medley, G.	1999	2	2				5.0	2.4	7.6
Rohrbach, D., Lechner, W., Ipinge, S., and Monyo, E.	1999	1	1	50.0	50.0	50.0			
Yapi, A., Debrah, S., Dehala, G., and Njomaha, C.	1999	2	2	85.0	75.0	95.0			
Yapi, A., Dehala, G., Ngawara, K., and Issaka, A.	1999	1	1	95.0	95.0	95.0			
Esterhuizen, J.	2000	4	4	89.5	64.0	119.0			
Randela, R.	2000	1	1				0.8	0.8	0.8
Schimmelpfennig, D., Thirtle, C., Van Zyl, J., Arnade, C., and Khatri, Y.	2000	2	2	51.0	44.0	58.0			
Falconi, C., Omamo, S., D'Ieteren, G., and Iraqi, F.	2001	1	2	32.0	32.0	32.0	5.1	5.1	5.1
Kaliba, A., Fox, S., and Norman, D.	2001	3	3				362.6	268.7	456.8
Rutherford, A., Odero, A., and Kruska, R.	2001	1	1				0.0	0.0	0.0
Townsend, R., and Thirtle, C.	2001	1	1	35.0	35.0	35.0			
Zeddies, J., Schaab, R., Neuenschwander, P., and Herren, H.	2001	1	1				199.0	199.0	199.0
Zegeye, T., Tesfahun, G., and Anandajayasekeram, P.	2001	1	1	29.0	29.0	29.0			
Bokonon-Ganta, A., De Groote, H., and Neuenschwander, P.	2002	1	1				145.0	145.0	145.0
Diaz-Hermelo, F., Langyintuo, A., and Lowenberg-DeBoer, J.	2002	1	1	4.7	4.7	4.7			

Table S-1 – Continued

Authors	Year	Number of		Internal Rate of Return			Benefit-Cost Ratio		
		Evaluations[a]	Estimates[a]	Average	Minimum	Maximum	Average	Minimum	Maximum
Kristjanson, P., Tarawali, S., Okike, I., Singh, B., Thornton, P., and Manyong, V.	2002	1	2	71.0	71.0	71.0	63.2	63.2	63.2
Mather, D., Bernsten, R., and Maredia, M.	2002	1	1	46.5	46.5	46.5			
Aw-Hassan, A., Shideed, K., Ceccarelli, S., Erskine, W., Grando, S., and Tutwiler, R.	2003	9	9	32.2	22.0	51.0			
De Groote, H., Ajuonu, O., Attignon, S., Djessou, R., and Neuenschwander, P.	2003	1	1				124.0	124.0	124.0
Johnson, N., Manyong, V., Dixon, A., and Pachico, D.	2003	2	2	15.5	9.0	22.0			
Johnson, N., Pachico, D., and Wortmann, C.	2003	1	1	18.0	18.0	18.0			
Thirtle, C., Lin, L., and Piesse, J.	2003	14	14	15.1	-12.0	58.0			
Nieuwoudt, W., and Nieuwoudt, T.	2004	1	2	17.0	17.0	17.0	1.6	1.6	1.6
Briones, R., Dey, M., Stobutzki, I., and Prein, M.	2005	24	24				29.0	1.3	100.0
Macharia, I., Lohr, B., and De Groote, H.	2005	1	2	86.0	86.0	86.0	24.0	24.0	24.0
Ajayi, O., Place, F., Kwesiga, F., and Mafongoya, P.	2007	3	3	13.1	3.2	20.8			
Boys, K., Faye, M., Fulton, J., and Lowenberg-DeBoer, J.	2007	1	1	13.3	13.3	13.3			
Dey, M., Kambewa, P., Prein, M., Jamu, D., Paraguas, F., and Pemsl, D.	2007	1	2	12.2	12.2	12.2	1.4	1.4	1.4
Laxmi, V., Erenstein, O., and Gupta, R.	2007	1	2	57.0	57.0	57.0	39.0	39.0	39.0
Briones, R., Dey, M., Ahmed, A., Prein, M., and Stobutzki, I.	2008	40	40				2.2	0.0	7.4
Fan, S., and Zhang, X.	2008	5	5				12.4	10.8	14.7
Akinola, A., Alene, A., Adeyemo, R., Sanogo, D., and Olanrewaju, A.	2009	8	16	31.1	17.0	43.0	11.0	3.0	24.0
Alene, A., and Coulibaly, O.	2009	27	27	45.9	5.0	82.0			
Alene, A., Menkir, A., Ajala, S., Olanrewaju, A., Manyong, V., and Ndiaye, A.	2009	10	20	48.8	28.0	74.0	23.0	10.0	84.0

Table S-1 – Continued

Authors	Year	Number of		Internal Rate of Return			Benefit-Cost Ratio		
		Evaluations[a]	Estimates[a]	Average	Minimum	Maximum	Average	Minimum	Maximum
Kaitibie, S., Omore, A., Rich, K., Salasya, B., Hooton, N., and Mwero, D.	2010	1	1	55.0	55.0	55.0			
Maredia, M., and Raitzer, D.	2010	1	2	4.0	4.0	4.0	1.0	1.0	1.0
Moussa, B., Lowenberg-DeBoer, J., Fulton, J., and Boys, K.	2011	8	8	59.7	8.3	132.3			
Macharia, I., Orr, A., Simtowe, F., and Asfaw, S.	2012	1	2	55.0	55.0	55.0	5.0	5.0	5.0
Magen, B.	2012	1	1	17.9	17.9	17.9			
Ayanwele, A., Adekunle, A., Akinola, A., and Adeyemo, V.	2013	6	12	30.5	22.0	38.0	24.2	9.2	44.0
Oleke, J., Manyong, V., Mignouna, D., Isinika, A., Mutabazi, K., and Hanna, R.	2013	1	2	13.2	13.2	13.2	1.0	1.0	1.0
Nedumaran, S., Bantilan, C., Mason-D'Croz, D., and Singh, P.	2014	1	1	50.0	50.0	50.0			

Source: Compiled by authors. See reference list below for further details.

[a] A single evaluation (of a particular technology, project, or program) within a given study may report multiple IRR or BCR estimates, or both

References

Abidogun, A. "Cocoa Research in Nigeria: An Ex-post Investment Analysis." *Nigerian Journal of Economics and Social Studies* 20 (1) (March 1978): 21-35.

Aghib, A.J. "The Economic Impact Assessment of the World Vision International - Purdue University Striga-Resistant Sorghum Initiatives." Purdue University, West Lafayette, Ind. U.S.A., 1996. Mimeo.

Ajayi, O.C., F. Place, F. Kwesiga, and P. Mafongoya. "Impacts of Improved Tree Fallow Technology in Zambia." Chapter in H. Waibel and D. Zilberman ed., *International Research on Natural Resource Management: Advances in Impact Assessment.* Wallingford, GB: Food and Agriculture Organization of the United Nations and Centre for Agricultural Bioscience International, 2007: 147-68.

Akgungor, S., D. Makanda, J. Oehmke, R. Myers, and Y. Choe. "Dynamic Analysis of Kenyan Wheat Research and Rate of Return." Contributed paper for the conference *Global Agricultural Science Policy for the Twenty-first Century*, Melbourne, August 26 to 28, 1996.

Akinola, A.A., A.D. Alene, R. Adeyemo, D. Sanogo, and A.S. Olanrewaju. "Economic impacts of soil fertility management research in west africa." *African Journal of Agricultural and Resource Economics* 3(2) (September 2009): 159-175.

Alene, A.D., and O. Coulibaly. "The Impact of Agricultural Research on Productivity and Poverty in Sub-Saharan Africa." *Food Policy* 34(2) (April 2009): 198-209.

Alene, A.D., A. Menkir, S.O. Ajala, B. Badu-Apraku, A.S. Olanrewaju, V.M. Manyong, and A. Ndiaye. "The Economic and Poverty Impacts of Maize Research in West and Central Africa." *Agricultural Economics* 40(5) (September 2009): 535-550.

Anandajayasekeram, P., D.R. Martella, J.H. Sanders, and B. Kuffuma. "Report on the Impact Assessment of the SADAC/ICRISAT Sorghum and Millet Improvement Program." Southern African Development Coordination Conference (SADCC) and Southern African Center for Cooperation in Agricultural Research (SACCAR), Ganorone, Botswana, 1995. Mimeo.

Anandajayasekeram, P., D.R. Martella, J. Sanders, B. Kupfuma. "Ex-ante analysis of the sorghum and millet improvement program". Chapter in Anandajayasekeram, P., M. Rukuni, S. Babu, F. Liebenberg, and C.L. Keswani ed., *Impact of Science on African Agriculture and Food Security*. Wallingford, U.K.: CABI Press, 2007: 57-67.

Arnade, C., D. Schimmelpfennig, C. Thirtle, and J. van Zyl. "Short and Long Run Returns to Agricultural R&D in South Africa, or Will the Real Rate of Return Please Stand Up!" Contributed paper for the conference *Global Agricultural Science Policy for the Twenty-first Century*, Melbourne, August 26 to 28, 1996.

Aw-Hassan, A., K. Shideed, S. Ceccarelli, W. Erskine, S. Grando, and R. Tutwiler. "The Impact of International and National Investment in Barley Germplasm Improvement in the Developing Countries." Chapter 11 in R.E. Evenson and D. Gollin ed., *Crop Variety Improvement and Its Effect on Productivity: The Impact of International Agricultural Research*. Wallingford, G.B.: Centre for Agricultural Bioscience International (CABI) Publishing, 2003: 241-256.

Ayanwale, A.B., A.A. Adekunle, A.A. Akinola, and V.A. Adeyemo. "Economic impacts of integrated agricultural research for development (IAR4D) in the Sudan Savanna of Nigeria." *African Development Review* 25 (1): 30-41.

Berlin, R. "Impact de la Recherche Agronomique: Le Cas de la Région de Segou, Mali." Swiss College of Agriculture, April 1997. Mimeo.

Bindlish, V., and R.E. Evenson. *Evaluation of the Performance of T&V Extension in Kenya*. World Bank Technical Paper N. 208. Washington, D.C.: World Bank, October 1993.

Bindlish, V. and R.E. Evenson. "The Impact of T&V Extension in Africa: The Experience of Kenya and Burkina Faso." *World Bank Research Observer* 12(2) (August 1997): 183-201.

Bindlish, V., R.E. Evenson, and M. Gbetibouo. *Evaluation of T&V Based Extension in Burkina Faso*. World Bank Technical Paper N. 226. Washington, D.C.: World Bank, November 1993.

Bokonon-Ganta, A.H., H. de Groote, and P. Neuenschwander. "Socio-Economic Impact of Biological Control of Mango Mealybug in Benin." *Agriculture, Ecosystems & Environment* 93(1)3 (2002): 367-378.

Boughton, D. and B.H. de Frahan. *Agricultural Research Impact Assessment: The Case of Maize Technology Adoption in Southern Mali*. MSU International Development Working Paper N. 41. East Lansing: Michigan State University, Department of Agricultural Economics, Department of Economics, 1994.

Boys, K., M. Faye, J. Fulton, and J. Lowenberg-DeBoer. "The Economic Impact of Cowpea Research in Senegal: An Ex-Post Analysis with Disadoption." *Agricultural Economics* 36(3) (May 2007): 363-375.

Briones, R.M., M.M. Dey, A.K.M. Mahfuzuddin Ahmed, M. Prein, and I. Stobutzki. "Priority Setting for Research on Aquatic Resources: An Application of Modified Economic Surplus Analysis to Natural Resource Systems." *Agricultural Economics* 39(2) (September, 2008): 231-43.

Briones, R., M. Dey, I. Stobutzki, and M. Prein. "Ex Ante Impact Assessment for Research on Natural Resources Management: Methods and Application to Aquatic Resource Systems." *Research Evaluation* 14(3) (December, 2005): 217-27.

Bua, A., G. Acola, R.L. Adupa, G.W. Otim-Nape, Y.K. Baguma, D. Sserunkuma, V. Manyong, O. Coulibaly. "The economic impact of investments in cassava research in Uganda". Chapter in Anandajayasekeram, P., M. Rukuni, S. Babu, F. Liebenberg, and CL Keswani ed., *Impact of Science on African Agriculture and Food Security*. Wallingford, UK: CABI Press. 2007.

Byerlee, D. and G. Traxler. "Economic Returns to National and International Wheat Improvement Research in the Post-Green Revolution Period." International Center for the Improvement of Maize and Wheat (CIMMYT), Mexico City, August 5, 1994. Mimeo.

Byerlee, D. and G. Traxler. "National and International Wheat Improvement Research in the Post-Green Revolution Period: Evolution and Impacts." *American Journal of Agricultural Economics* 77(2) (May 1995): 268-278.

Byerlee, D., and G. Traxler. "Estimation of Actual Spillovers of National and International Wheat Improvement Research." Chapter in M.K. Maredia and D. Byerlee ed., *The Global Wheat Improvement System: Prospects for Enhancing Efficiency in the Presence of Spillovers. CIMMYT Research Report N. 5*. Mexico: International Maize and Wheat Improvement Center (CIMMYT), 1999.

Chisi, M., P. Anandajayasekeram, D.R. Martella, M.M. Ahmed, M. Mwape. *Impact Assessment of Sorghum Research in Zambia, 1983-2010*. SACCAR, 84. Botswana: Southern African Center for Cooperation in Agriculrural and Natural Resources Research and Training and Southern African Development Community, 1996.

De Groote, H., O. Ajuonu, S. Attignon, R. Djessou, and P. Neuenschwander. "Economic Impact of Biological Control of Water Hyacinth in Southern Benin." *Ecological Economics* 45 (2003): 150-217.

Dey, M.M., P. Kambewa, M. Prein, D. Jamu, F.J. Paraguas, D.E. Pemsl, and R.M. Briones. "World Fish Centre. Impact of the Development and Dissemination of Integrated Aquaculture–Agriculture Technologies in Malawi." Chapter in H. Waibel and D. Zilberman ed., *International Research on Natural Resource Management: Advances in Impact Assessment*. Wallingford, GB: Food and Agriculture Organization of the United Nations and Centre for Agricultural Bioscience International, 2007: 118-46.

Diaz-Hermelo, F., A. Langyintuo, and J. Lowenberg-DeBoer. "Impact of Cowpea Breeding and Storage Research in Cameroon." Chapter in C.A. Fatokun, S.A. Tarawali, B.B. Singh, P.M. Kormawa and M. Tambo ed., *Challenges and Opportunities for Enhancing Sustainable Cowpea Production*. Ibadan, Nigeria: International Institute of Tropical Agriculture, 2002: 407–423.

Edwin, J. and W.A. Masters. "Returns to Rice Technology Development in Sierra Leone." Department of Agricultural Economics, Purdue University, West Lafayette, March 1997. Mimeo.

Elbasha, E., P.K. Thornton, and G. Tarawali. *An Ex Post Economic Impact Assessment of Planted Forages in West Africa*. ILRI Impact Assessment Series 2. Nairobi, Kenya: International Livestock Research Institute, 1999.

Esterhuizen, J.M.C., C.W. Stoltsz, C.J. van Rooyen, and L. D'Haese. "An impact analysis of the biological control programme of invasive Prosopis species in the Britstown-de AAR district." *Agrekon* 40(4) (December 2001): 728-737.

Evenson, R.E. *The International Agricultural Research Centers: Their Impact on Spending for National Agricultural Research and Extension Consultative Group on International Agricultural Research (CGIAR).* Study Paper N. 22. Washington, DC: The World Bank, 1987.

Evenson, R.E. and P. Flores. "Social Returns to Rice Research." Chapter in IRRI ed., *Economic Consequences of the New Rice Technology*. Los Baños, Philippines: International Rice Research Institute (IRRI), 1978: 243-65.

Evenson, R.E. and Y. Kislev. *Agricultural Research and Productivity*. Ithaca, New York: Yale University Press, 1975.

Falconi, C.A., S.W. Omamob, G. d'Ieteren, and F. Iraq. "An Ex Ante Economic and Policy Analysis of Research on Genetic Resistance to Livestock Disease: Trypanosomosis in Africa." *Agricultural Economics* 25(2)3 (September 2001): 153-163.

Fan, S., and X. Zhang. "Public Expenditure, Growth and Poverty Reduction in Rural Uganda." *African Development Review* 20(3) (2008): 466-496.

Howard, J. *The Economic Impact of Improved Maize Varieties in Zambia*. PhD dissertation, Michigan State University, East Lansing, 1994.

Howard, J., G.M. Chitula, and S.M. Kalonge. *The Impact of Investments in Maize Research and Dissemination in Zambia, Part I: Main Report, and Part II: Annexes*. International Development Working Paper N. 39/2. East Lansing: Michigan State University, Department of Agricultural Economics, Department of Economics, 1993.

Howard, J. and C. Mungoma. *Zambia's Stop-and-Go Revolution: The Impact of Policies and Organizations on the Development and Spread of Maize Technology*. MSU International Development Working Paper N. 61. East Lansing: Michigan State University, Department of Agricultural Economics, Department of Economics, 1996.

Isinika, Aida C. *Assessing the Effect of Agricultural Research Expenditures on Agricultural Productivity in Tanzania*. PhD Dissertation, University of Kentucky, Lexington, 1997.

Johnson, N.L., V.M. Manyong, A.G.O. Dixon, and D. Pachico. "The Impact of IARC Genetic Improvement Programmes on Cassava." Chapter in R.E. Evenson and D. Gollin ed., *Crop Variety Improvement and Its Effect on Productivity: The Impact of International Agricultural Research*. Wallingford, G.B.: Centre for Agricultural Bioscience International (CABI) Publishing, 2003: 337-356.

Johnson, N.L., D.H. Pachico, and C.S. Wortmann. "The Impact of CIAT's Genetic Improvement Research on Beans."Chapter in R.E. Evenson and D. Gollin ed., *Crop Variety Improvement and Its Effect on Productivity: The Impact of International Agricultural Research*. Wallingford, G.B.: Centre for Agricultural Bioscience International (CABI) Publishing, 2003: 257-274.

Kaitibie, S., A. Omore, K. Rich, and P. Kristjanson. "Kenyan Dairy Policy Change: Influence Pathways and Economic Impacts." *World Development* 38(10) (2010): 1494–1505.

Kaliba, A., S. Fox, and D. Norman. "Economic returns from livestock research and development in Tanzania: 1966-1995". Chapter in Anandajayasekeram, P., M. Rukuni, S. Babu, F. Liebenberg, and C.L. Keswani ed., *Impact of Science on African Agriculture and Food Security*. Wallingford, U.K.: Center for Agriculture and Bioscience International (CABI) Press. 2007.

Karanja, D. *The Rate of Return to Maize Research in Kenya: 1955-88*. MSc thesis, Michigan State University, East Lansing, 1990.

Karanja, D. *An Economic and Institutional Analysis of Maize Research in Kenya*. MSU International Development Working Paper No. 57. East Lansing: Michigan State University, Department of Agricultural Economics, Department of Economics, 1996.

Khatri, Y.J., C.G. Thirtle, and J. van Zyl. "Public Research and Development as a Source of Productivity Change in South African Agriculture." *South African Journal of Science* 92(3) (1996): 143-150.

Kristjanson, P.M., B.M. Swallow, G.J. Rowlands, R.L. Kruska, and P.N. de Leeuw. "Measuring the Costs of African Animal Trypanosomosis, the Potential Benefits of Control and Returns to Research." *Agricultural Systems* 59(1) (1999): 79-98.

Kristjanson, P.,S. Tarawali, I. Okike, B.B. Singh, P.K. Thornton, V.M. Manyong, R.L. Kruska, and G. Hoogenboom. *Genetically Improved Dual-Purpose Cowpea: Assessment of Adoption and Impact in the Dry Savannah Region of West Africa*. ILRI Impact Assessment Series No. 9. International Livestock Research Institute (ILRI) and International Institute of Tropical Agriculture (IITA), 2002.

Kristjanson, P.M., E. Zerbini, K.P.C. Rao, V. Kiresur, and P. Hofs. *Genetic Enhancement of Sorghum and Millet Residues Fed to Ruminants: An Ex Ante Assessment of Returns to Research*. ILRI Impact Assessment Series 3. Nairobi, Kenya: International Livestock Research Institute (ILRI), 1999.

Kupfuma, B. *The Payoffs to Hybrid Maize Research in Zimbabwe: An Economic and Institutional Analysis*. MSc thesis, Michigan State University, East Lansing, 1994.

Laker-Ojok, R. *The Rate of Return to Agricultural Research in Uganda: The Case of Oilseeds and Maize*. MSU International Development Working Paper N. 42. East Lansing: Michigan State University, Department of Agricultural Economics, Department of Economics, 1994a.

Laker-Ojok, R. *The Potential Returns to Oilseeds Research in Uganda: The Case of Groundnuts and Sesame*. MSU International Development Working Paper N. 45. East Lansing: University of Michigan, Department of Agricultural Economics, Department of Economics, 1994b.

Laxmi, V., O. Erenstein, and R.K. Gupta. "CIMMYT Assessing the Impact of Natural Resource Management Research: The Case of Zero Tillage in India's Rice–Wheat Systems." Chapter in H. Waibel and D. Zilberman ed., *International Research on Natural Resource Management: Advances in Impact*

Assessment. Wallingford,G.B.: Food and Agriculture Organization (FAO) of the United Nations and Centre for Agricultural Bioscience International (CABI), 2007: 68-90.

Lubulwa, G. *The Human Health Benefits of Research to Reduce the Hydrogen Cyanide Potential in Cassava Cultivars in Africa -- A Completed Project Assessment of ACIAR Project PN9007*. Economic Evaluation Unit Working Paper No. 21. Canberra: Australian Centre for International Agricultural Research (ACIAR), May 1995.

Lubulwa, G., D. Gwaze, J. Clarke, P. Milimo, and J. Mulutya. *Overcoming the Shortage of Fuelwood and Poles Through Forestry Research: Estimates of Benefits from Three Complete ACIAR Forestry Projects in Africa and Thailand*. Economic Evaluation Unit Working Paper No. 22. Canberra: Australian Centre for International Agricultural Research (ACIAR), June 1995.

Lubulwa, G., B. Wafula, E. Craswell, I. Willett, and J. Davis. *Dry Land Farming in the Semi-Arid Tropics of Kenya: ACIAR Project Experience*. Economic Evaluation Unit Working Paper No. 19. Canberra: Australian Centre for International Agricultural Research (ACIAR), May 1995.

Macharia, I., B. Löhr, and H. De Groote. "Assessing the potential impact of biological control of plutella xylostella (diamondback moth) in cabbage production in Kenya." *Crop Protection* 24(11) (November 2005): 981-989.

Macharia, I., A. Orr, F. Simtowe, and S. Asfaw. "Potential economic and poverty impact of improved chickpea technologies in Ethiopia." Presented at the *International Asociation of Agricultural Economists Conference*, Foz do Iguacu, Brasil, August 18 to 24, 2012.

MacMillan, J.A., G. Mudimu, J.F. MacRobert, L. Rugube, E. Guveya, L.T. Mutemeri, K. Chakanyuka, and B. Johnston. "Measuring Benefits and Costs of Smallholder Maize Extension Research, Zimbabwe." University of Manitoba, Manitoba: March 1992. Mimeo.

Magen, B.B. *An Ex Post Economic Impact Assessment of Bean/Cowpea Crsp's Investment on Varietal Development in Senegal*. MSU No. 142739. East Lansing, Michigan: Michigan State University, Department of Agricultural, Food , and Resource Economics, 2012.

Makanda, D.W., J.F. Oehmke. "Economics of wheat research in Kenya". Chapter in P. Anandajayasekeram, M. Rukuni, S. Babu, F. Liebenberg, and C.L. Keswani ed., *Impact of Science on African Agriculture and Food Security*. Wallingford, U.K.: Center for Agriculture and Bioscince International (CABI) Press, 2007.

Marasas, C.N. "Impact of the russian wheat aphid integrated control programme in south Africa". Chapter in P. Anandajayasekeram, M. Rukuni, S. Babu, F. Liebenberg, and C.L. Keswani ed., *Impact of Science on African Agriculture and Food Security*. Wallingford, U.K.: Center for Agriculture and Bioscince International (CABI) Press, 2007: 91-102.

Maredia, M.K., and D. Byerlee. "The Efficiency of Wheat Improvement Research Investments in the Presence of Spillins." Chapter in M.K. Maredia and D. Byerlee ed., *The Global Wheat Improvement System: Prospects for Enhancing Efficiency in the Presence of Spillovers. CIMMYT Research Report No. 5*. Mexico: International Maize and Wheat Improvement Center (CIMMYT), 1999.

Maredia, M.K., and D.A. Raitzer. "Estimating Overall Returns to International Agricultural Research in Africa through Benefit-Cost Analysis: A "Best-Evidence" Approach." *Agricultural Economics* 41(1) (January 2010): 81-100.

Mather, D., R.H. Bernsten, and M.K. Maredia. *Ex-Ante Assessment of the Impact of Disease-Resistant Cucurbits Germplasm in Indonesia and South Africa*. MSU No. 11542. East Lansing, Michigan: Michigan State University, Department of Agricultural, Food, and Resource Economics, 2002.

Mazhangara, E., P. Anandajayasekeram, M.P. Mudhara, D.R. Martella, and M. Murata. *Impact Assessment of Groundnut Research and the Enabling Environment in Zimbabwe: 1960-2000*. Gaborone, Botswana: Southern African Centre for Cooperation in Agricultural Research (SACCAR) and Natural Resource Research Training, 1997.

Mazzucato, V. *Non-Research Policy Effects on the Rate of Return to Maize Research in Kenya: 1955-1988*. MSc thesis, Michigan State University, East Lansing, 1991.

Mazzucato, V. and S. Ly. *An Economic Analysis of Research and Technology Transfer of Millet, Sorghum, and Cowpeas in Niger*. Discussion Paper No. 93-06. The Hague: International Service for National Agricultural Research, February 1993.

Mokoena, M.R. "Impact of investments in livestock research and development programmes in South Africa." Chapter in P. Anandajayasekeram, M. Rukuni, S. Babu, F. Liebenberg, and C.L. Keswani ed., *Impact of Science on African Agriculture and Food Security*. Wallingford, U.K.: Center for Agriculture and Bioscience International (CABI) Press, 2007: 236-248.

Mokoena, M.R., R.F. Townsend, and J.F. Kirsten. "Cattle Improvement Schemes in South Africa: Measuring the Returns to Research Investments."*Agrekon: Agricultural Economics Research, Policy and Practice in Southern Africa* 38(1) (March 1999): 1-12.

Moshi, A.J., P. Anandajayasekeram, A. Kaliba, D. Martella, W. Mwangi, and F.M. Shao. "Economic impact of maize research in Tanzania". Chapter in P. Anandajayasekeram, M. Rukuni, S. Babu, F. Liebenberg, and C.L. Keswani ed., *Impact of Science on African Agriculture and Food Security*. Wallingford, U.K.: Center for Agriculture and Bioscience International (CABI) Press, 2007: 74-82.

Moussa, B., J. Lowenberg-DeBoer, J. Fultona, and K. Boys. "The Economic Impact of Cowpea Research in West and Central Africa: A Regional Impact Assessment of Improved Cowpea Storage Technologies." *Journal of Stored Products Research* 47 (March, 2011): 147-156.

Mudhara, M.P., P. Anandajayasekeram, B. Kupfuma, and E. Mazhangara. *Impact Assessment of Cotton Research and the Enabling Environment in Zimbabwe 1970-1995* . Gaborone, Botswana: Southern African Centre for Cooperation in Agricultural Research (SACCAR), 1995.

Mukhebi, A.W., T. Chamboko, C.J. O'Callaghan, T.F. Peter, R.L. Kruska, G.F. Medley, S.M. Mahan, and B.D. Perry. "An Assessment of the Economic Impact of Heartwater (Cowdria Ruminantium Infection) and Its Control in Zimbabwe." *Preventive Veterinary Medicine* 39(3) (1999): 173-189.

Murata, M., P. Anandajayasekeram, M.P. Mudhara, D.R. Martella, and E. Mazhangara. *Impact Assessment of Sunflower Research and the Enabling Environment in Zimbabwe, 1976-2000*. Gaborone, Botswana: Southern African Centre for Cooperation in Agricultural Research (SACCAR), 1997.

Nedumaran, S., C. Bantilan, D. Mason-D'Croz, and P. Singh. "Application of multi-commodity partial equilibrium model to quantify the welfare benefits of research." Presented at *Australian Agricultural and Resource Economics Society 58th Conference*, Port Maquarie, Australia, February 4 to 7, 2014.

Niederwieser, J.G., P. Anandajayasekeram, M. Coetzee, D. Martella, B. Pieterse, and C. Marasas. *Socio-Economic impact of the Lachenalia research program*. Gaborone, Botswana: Southern African Centre for Cooperation in Agricultural Research (SACCAR), 1997.

Nieuwoudt, W.L., and T.W. Nieuwoudt. "The rate of return on R&D in the south African sugar industry, 1925–2001." *Agrekon* 43(3) (2004): 265-275.

Njomaha, C., A. Adamou, and A.M. Yapi. "Etude d'Impact de la S 35 dans l'Extreme-Nord Cameroun." December 1996. Mimeo.

Norgaard, R. "The Biological Control of Cassava Mealybug in Africa." *American Journal of Agricultural Economics* 70(2) (May 1988): 366-371.

Oleke, J.M., V. Manyong, D. Mignouna, A. Isinika, K. Mutabazi, R. Hanna, and M. Sabelis. "Ex-ante economic analysis of biological control of coconut mite in Benin." *AgBioForum* 16(2) (2013): 161-169.

Ouedraogo, S. and M.K. Bertelsen. "The Value of Research on Indigenous Knowledge: Preliminary Evidence from the Case of Zai in Burkina Faso." *Journal of Sustainable Agriculture* 10(1) (April 1997): 33-42.

Ouedraogo, S. and L. Illy. "Evaluation de l'Impact Economique des Cordons Pierreux: Cas du Plateau Central au Burkina Faso." Institut d'Etudes et de Recherches Agricoles, Ouagadougou, April 1996. Mimeo.

Ouedraogo, S., L. Illy, and F. Lompo. "Evaluation de l'Impact Economique de la Recherche et la Vulgarisation Agricole: Cas du Mais dans l'Ouest du Burkina Faso." Institut d'Etudes et de Recherches Agricole, Ouagadougou, May 1995. Mimeo.

Randela, R. *Socio-economic Impact Analysis of Livestock Disease Control Programmes, with Special Reference to Tick and Tick-borne Diseases*. MSc thesis, University of Pretoria, Pretoria, South Africa, 2000.

Rohrbach, D.D., W.R. Lechner, S.A. Ipinge, and E.S. Monyo. *Impact from Investments in Crop Breeding: The Case of Okashana 1 in Namibia*. Impact Series No. 4. Andhra Pradesh, India: International Crops Research Institute for the Semi-Arid Tropics (ICRISAT), 1999.

Rueda, J.L., P.T. Elwell, T.S. Walker, M. Soto, M. Bicamumpaka, and D. Berrios. "Economic Impact of High-Yielding, Late-Blight-Resistant Varieties in the Eastern and Central African Highlands." Chapter in T.S. Walker and C.C. Crissman ed., *Case Studies of the Economic Impact of CIP-Related Technology*. Lima: International Potato Center (CIP), 1996: 15-30.

Rutherford, A.S., A.N. Odero, and R.L. Kruska. *The Role of the Broadbed Maker Plough in Ethiopian Farming Systems: An Ex Post Impact Assessment Study*. ILRI Impact Assessment Series 7. Nairobi, Kenya: International Livestock Research Institute (ILRI), 2001.

Sanders, J.H., T. Bezuneh, and A. Schroeder. *Impact Assessment of the SAFGRAD Commodity Networks*. Washington, D.C.: U.S. Agency for International Development, January 1994.

Schimmelpfennig, D., C. Thirtle, J. van Zylc, C. Arnaded, and Y. Khatri. "Short and Long-Run Returns to Agricultural R&D in South Africa, or Will the Real Rate of Return Please Stand Up?" *Agricultural Economics* 23(1) (June 2000): 1-15.

Schwartz, L.A., J.A. Sterns, and J.F. Oehmke. "Economic Returns to Cowpea Research, Extension, and Input Distribution in Senegal." *Agricultural Economics* 8(2) (February 1993): 161-171.

Schwartz, L.A., J.A. Sterns, J.F. Oehmke, and R.D. Freed. "Impact Study of the Bean/Cowpea CRSP for Senegal." November 1989. Mimeo.

Seck, P.A., M. Sidibe, and A.M. Beye. "Impact Social de la Recherche et du Transfert de Technologies sur le Coton au Sénégal." Institut Sénégalais de Recherche Agricole, July 1995. Mimeo.

Seidi, S. "An Economic Analysis of Mangrove Rice Research, Extension and Seed Production in Guinea-Bissau: Preliminary Evidence from the Tombali Region." Purdue University, Department of Agricultural Economics, West Lafayette, 1997. Mimeo.

Smale, M. and P.W. Heisey. "Maize Research in Malawi Revisited: An Emerging Success Story?" *Journal of International Development* 6(6) (November/December 1994): 689-706.

Sterns, J.A. and R.H. Bernsten. *Assessing the Impact of Cowpea and Sorghum Research and Extension in Northern Cameroon*. MSU International Development Working Papers No. 43. East Lansing: Michigan State University, Department of Agricultural Economics, Department of Economics, 1994.

Thirtle, C., J. Atkins, P. Bottomley, N. Gonese, and J. Govereh. "Agricultural Research and Productivity; the Green Revolution in Zimbabwe." Presented for the *ESRC Development Economics Study Group Annual Conference*, March, 1992.

Thirtle, C., J. Atkins, P. Bottomley, N. Gonese, J. Govereh, and Y. Khatri. "Agricultural Productivity in Zimbabwe, 1970-90." *Economic Journal* 103(417) (March 1993): 474-480.

Thirtle, C., L. Lin, and J. Piesse. "The Impact of Research-Led Agricultural Productivity Growth on Poverty Reduction in Africa, Asia and Latin America." *World Development* 31(12) (December 2003): 1959-1975.

Thirtle, C.G., H.S. von Bach, and J. van Zyl. "Explaining Total Factor Productivity Growth in South African Commercial Agriculture, 1947-91." Working Paper No. 93/02, University of Reading, Department of Agricultural Economics and Management, March 1993.

Townsend, R., and C. Thirtle. "Is Livestock Research Unproductive? Separating Health Maintenance from Improvement Research." *Agricultural Economics* 25(2)3 (September 2001): 1771-89.

Townsend, R., and J. van Zyl. "Estimation of the rate of return to wine grape research and technology development expenditures in South Africa/Bepaling van die opbrengskoers van navorsing en tegnologie-ontwikkeling in die wyndruifindustrie." *Agrekon* 37(2): 189-210.

Townsend, R.F., J. van Zyl, and C. Thirtle. "Assessing the benefits of research expenditures on maize production in South Africa/Raming van die voordele van navorsingsbestedings op mielieproduksie in Suid-Afrika." *Agrekon* 36(4): 585-597.

Tré, J-P. *The Rates of Return to Mangrove Rice Research in West Africa*. MSc thesis, Purdue University, West Lafayette, 1995.

Wessels, J., P. Anandajayasekeram, C.J. Van Rooyen, C. Marasas, G. Littlejohn, and C. Coetzee. "Does research and development pay-the case for Proteaceae". *Agrekon* 37 (4) (1998): 601-611.

Yallah, N. "Evaluation de l'Impact de la Recherche Agronomique au Tchad: Caves du Coton." Institut du Sahel, Ministère du Développement Rural, Tchad, August 1996. Mimeo.

Yapi, A.M., S.K. Debrah, G. Dehala, and C. Njomaha. *Impact of Germplasm Research Spillovers: The Case of Sorghum Variety S 35 in Cameroon and Chad*. Impact Series No. 3. Andhra Pradesh, India: International Crops Research Institute for the Semi-Arid Tropics (ICRISAT), 1999.

Yapi , A.M., G. Dehala, K. Ngawara, and A. Issaka. *Assessment of the Economic Impact of Sorghum Variety S35 in Chad*. Impact Series No. 6. Andhra Pradesh, India: International Crops Research Institute for the Semi-Arid Tropics (ICRISAT), 1999.

Yapi, A.M., A.O. Kergna, S.K. Debrah, A. Sidibe, and O. Sanogo. "Analyse Economique de l'Impact de la Recherch sur le Sorgho et le Mil au Mali." International Crops Research Institute for the Semi-Arid Tropics, Bamako, December 1996. Mimeo.

Zeddies, J., R.P. Schaab, P. Neuenschwander, and H.R. Herren. "Economics of Biological Control of Cassava Mealybug in Africa." *Agricultural Economics* 24(2) (2001): 209-219.

Zegeye, T., G. Tesfahun, P. Anandajayasekeram. "Impact of maize technology development and transfer in Ethiopia". Chapter in P. Anandajayasekeram, M. Rukuni, S. Babu, F. Liebenberg, and C.L. Keswani ed., *Impact of Science on African Agriculture and Food Security*. Wallingford, U.K.: Centre for Agriculture and Bioscience International (CABI) Press, 2007: 116-126.
